# Effect of HPV Infection on the Occurrence and Development of Laryngeal Cancer: A Review

**DOI:** 10.7150/jca.34016

**Published:** 2019-07-23

**Authors:** Dongli Yang, Yong Shi, Yemei Tang, Hongyu Yin, Yujia Guo, Shuxin Wen, Binquan Wang, Changming An, Yongyan Wu, Wei Gao

**Affiliations:** 1 Shanxi Key Laboratory of Otorhinolaryngology Head and Neck Cancer, First Hospital of Shanxi Medical University, Taiyuan 030001, Shanxi, China.; 2 Department of Otolaryngology Head & Neck Surgery, First Hospital of Shanxi Medical University, Taiyuan 030001, Shanxi, China.; 3 The Key Scientific and Technological Innovation Platform for Precision Diagnosis and Treatment of Head and Neck Cancer, Shanxi Province, Taiyuan 030001, Shanxi, China.; 4 Department of Head and Neck Surgery, Cancer Hospital, National Cancer Center, Chinese Academy of Medical Sciences & Peking Union Medical College, Beijing 100021, China.

**Keywords:** Human Papillomavirus, HPV Testing, Incidence, Head and Neck Carcinoma, Laryngeal Carcinoma

## Abstract

Laryngeal cancer has the second highest incidence of head and neck malignant tumors worldwide. In recent years, studies have shown that human papillomavirus (HPV) infection may be a high-risk factor for laryngeal cancer and closely related to the development and prognosis of laryngeal cancer. The mechanism of the occurrence and development of laryngeal cancer caused by HPV infection needs investigation, as does a rapid and effective HPV detection method for effectively preventing the occurrence of laryngeal cancer and controlling its development. Many studies have explored the relation between HPV infection and laryngeal cancer. Here we review the research progress in investigating HPV infection in terms of DNA, mRNA and protein levels in the occurrence and development of laryngeal cancer and routine HPV detection methods.

## Introduction

Laryngeal cancer is the most common head and neck malignant tumor in the northern areas of China and its main pathological type is laryngeal squamous cell carcinoma (LSCC) 1. In recent years, the incidence of LSCC has been increasing, seriously threatening the life and health of humans. Laryngeal cancer can result from the combined action of smoking, drinking, air pollution, HPV infection and sex hormone levels. Recently, increasing studies have found that among the many pathogenic factors of laryngeal cancer, high-risk HPV (HR-HPV) infection may be one of the most important 2, 3. Epidemiological studies reported HPV infection rates in laryngeal cancer ranging from 8% to 83% 3, 4; a meta-analysis showed a 28% HPV infection rate in all LSCC 3, and HPV infection was correlated with its occurrence 5. The infection rate of HPV virus was relatively higher in Chinese people than Europeans and North Americans with laryngeal cancer 6, and there may be a strong link between high-risk HPV infection and risk of laryngeal cancer in Chinese people with laryngeal cancer. However, other studies have not found HPV infection affecting the occurrence and development of laryngeal cancer or prognosis. For example, in a study of 674 patients with laryngeal cancer in China, HPV infection prevalence was only 4.9% in laryngeal cancer patients 7, and HPV infection was not a major risk factor for laryngeal cancer. Table 1 shows some of the results of research of laryngeal cancer patients.

These differences in findings may be due to a variety of factors such as differences in population geographical distribution, test sample quality, and sensitivity and specificity of test methods used. However, they are mainly attributed to the unclear molecular mechanism of laryngeal cancer caused by HPV infection and different detection methods. This paper reviews the effects of HPV infection on the development of laryngeal cancer and the advantages and disadvantages of routine HPV detection methods in terms of DNA, mRNA and protein levels.

## The mechanism of HPV infection affecting the occurrence and development of laryngeal cancer

**Figure 1:**
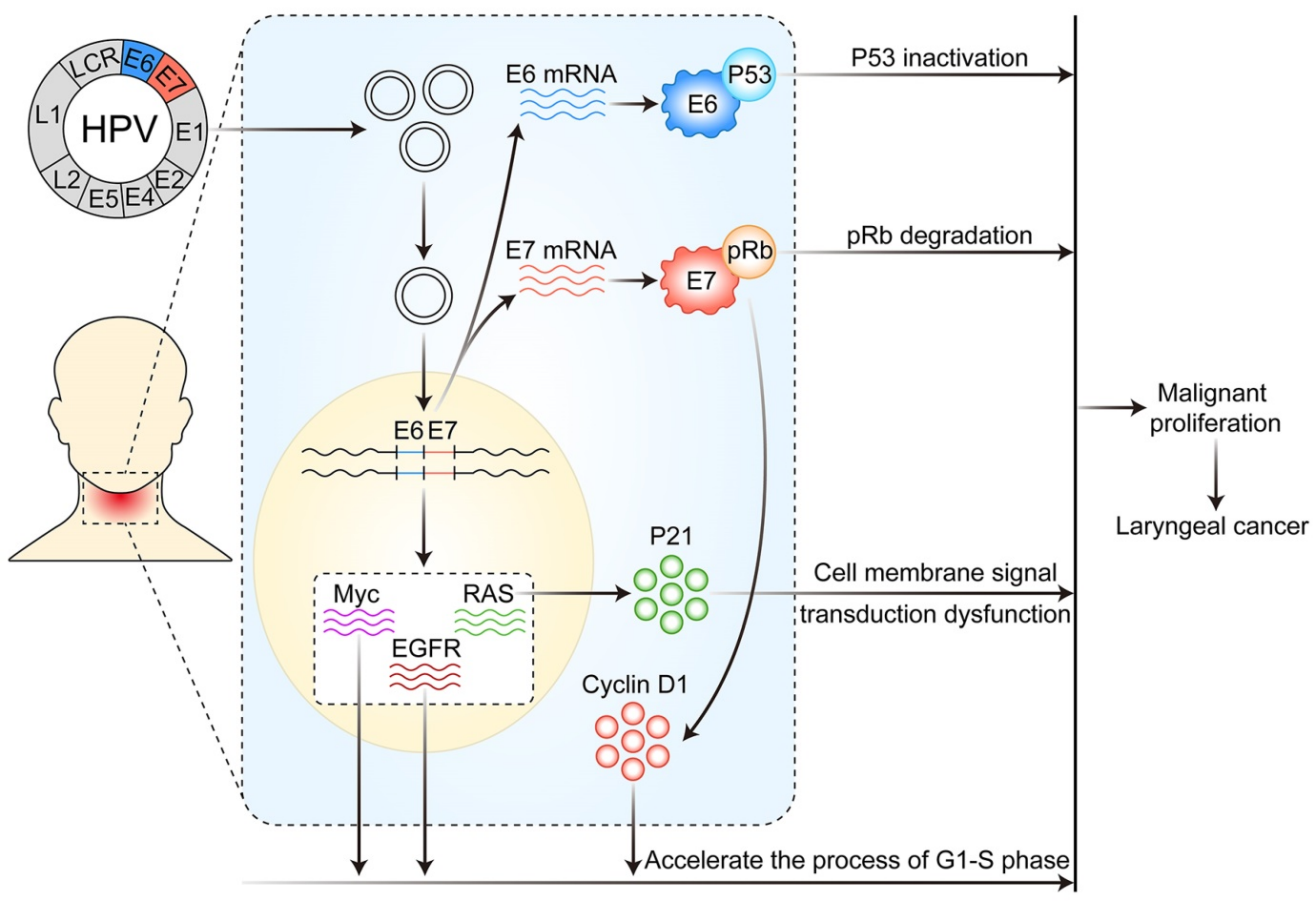
Mechanism of HPV infection affecting the occurrence and development of laryngeal cancer. After HPV infects laryngeal mucosal epithelial cells, its DNA is integrated into the host cell genome, thereby resulting in altered expression of a variety of proto-oncogenes and tumor suppressor genes, the overexpression of E6 and E7 mRNA and altered function of various proteins in host cells, thus leading to disordered cell metabolism and malignant proliferation.

As early as 1982, Syrijanen et al.^28^ suggested that HPV infection was closely associated with laryngeal cancer. After then, many studies have shown that infection with HR-HPV (especially HPV16 and 18) and the expression of the early coding genes E6 and E7 may be important risk factors for the occurrence of laryngeal cancer 29, 30. The possible mechanism of HPV infection affecting the development and progression of laryngeal cancer is described at the following three levels: DNA, mRNA and protein. (Figure 1)

### HPV infection affects the development of laryngeal cancer via genomic integration, activating proto-oncogenes and inhibiting anti-oncogenes

The occurrence and development of human cancer is closely related to the selective expression of genes in time or space (including the expression of normal or mutant genes and the expression of different amounts of genes). HPV infection mainly affects the occurrence and development of laryngeal cancer by integrating in the host cell genome, activating the expression of proto-oncogenes and inhibiting the expression of tumor suppressor genes. Its DNA enters the nucleus in a free state, and then integrates into the genome of the host cells as soon as HPV infects via the damaged laryngeal mucosa epithelial cells to the host cells. The integration of the HPV DNA can lead to genetic mutation and expression disorder of host cells, which leads to cell metabolic disorder and malignant proliferation.

When HPV infects the larynx, it can promote the development of laryngeal cancer by activating the expression of various proto-oncogenes. For instance, in the larynx, HPV infection may cause excessive expression or mutation of the proto-oncogene RAS, which may lead to a cell-membrane signal transduction disorder mediated by its protein product p21, thereby resulting in malignant cell proliferation and laryngeal carcinoma. HPV infection can also activate the expression of epidermal growth factor receptor and Myc genes, promoting cells moving from the G1 to S phase, thus accelerating the cell cycle and leading to laryngeal cancer.

HPV infection causes laryngeal cancer, which is also associated with tumor suppressor gene mutation and inactivation. p53, Rb and p16 genes are all important tumor suppressor genes in the body and participate in regulating cell proliferation cycle. After HPV infects the larynx, early E6 gene expression can cause p53 inactivation and E7 expression can cause Rb inactivation 31, which leads to cell cycle disorder, uncontrolled proliferation, and eventually malignant tumors. At the same time, HPV infection leading to p16 inactivation plays an important role during early laryngeal cancer 32.

HPV infection can also be carcinogenic by interacting with the apoptotic inhibiting gene, survivin, cyclin D1 and human telomere reverse transcriptase, thus accelerating cell cycle progression, inhibiting cell senescence, and promoting tumorigenesis 33-35.

### High mRNA expression of HPV affects the expression of genes related to the proliferation and migration of laryngeal carcinoma

After HPV enters laryngeal mucosa epithelial cells, HPV DNA integrates in the host cell genome to cause gene mutation and expression disorder and transcribes and expresses the viral oncoprotein to cause cancer. Fusconi et al.^36^ indicated that if HPV DNA is detected in LSCC patients, HPV mRNA levels must be detected, because the presence of HPV DNA in LSCC is not sufficient to prove its carcinogenesis. This suggestion might indicate that transient infection of HPV is not related to the carcinogenic process 36, 37. The mRNA expression of HPV can indicate that tumor transformation is caused by HPV, although this postulation does not exclude that other carcinogens may also play a role in the carcinogenic effect.

The mRNA expression of HPV is increased with the development of laryngeal lesions (vocal polyps, lesions before larynx cancer, laryngeal cancer), and the mRNA expression of HPV is also increased 15. The mRNA expression of HPV in laryngeal cancer tissues was found abnormally increasedand related to the expression of genes involved in proliferation and migration of laryngeal cancer cells 38. Therefore, the mRNA expression of HR-HPV plays an important role in the development and progression of laryngeal cancer. The detection of HPV mRNA can provide important clinical guidance for preventing and monitoring the development of laryngeal cancer.

Poltronieri et al. found that HR-HPV genome transcribes viral miRNAs in the HPV infection and cancer development process, and miRNA silencing could be a valuable approach to support pharmaceutical interventions in HPV-dependent cancers 39. This approach may also be a new treatment for HPV-associated laryngeal cancer.

### The association between HPV E6, E7 protein and related proteins of cell cycle regulation and carcinogenesis in laryngeal cancer

HPV infection induces laryngeal cancer mainly because of excessive expression of HR-HPV E6 and E7 proteins. When HPV infects laryngeal tissues, it can cause excessive expression of HPV E6 and E7 proteins and directly or indirectly affect the stability and activity of proto-oncogenes and tumor suppressor genes. For example, HPV16 E7 protein can activate the cell cycle regulation protein D1 (cyclin D1), thereby accelerating the G1-to-S phase in cells and leading to the occurrence and development of laryngeal cancer 40. Zeng et al.^41^ also supported this view and pointed out the association of positive expression of HPV16 E7 and cyclin D1 with laryngeal cancer clinical staging (I-IV), with a significant positive correlation between their expression and the incidence of laryngeal cancer. This finding indicates that the occurrence and development of laryngeal cancer is closely related to the overexpression of HPV16 E7 protein and cyclin D1. Therefore, the expression of HPV16 E7 and cyclin D1 must be detected in laryngeal cancer tissues for early diagnosis and early treatment of laryngeal cancer, and both can also be used as biomarkers to determine the malignant degree and prognosis of laryngeal cancer 42.

Studies have shown that after HR-HPV16 and 18 infect the larynx, the early gene product E6 protein can bind with p53 protein and form a complex resulting in p53 inactivation and thus hindering the role of p53 protein in tumor inhibition in cells. The HR-HPV16 E7 protein can bind to the Rb gene product, thus degrading the Rb protein so it loses its function of inhibiting cell proliferation 31.

Highly expressed cyclin D1 and high-risk p53 mutations were found associated with poor disease-specific survival rate in patients with laryngeal cancer. The functional classification of p53 mutants based on EAp53 can predict the disease-specific survival rate with advanced laryngeal cancer and p53 mutation. The model of Bcl-xL and cyclin D1 staining, p53 mutation classification and HPV status is of great significance for predicting disease-specific survival 43.

**Figure 2:**
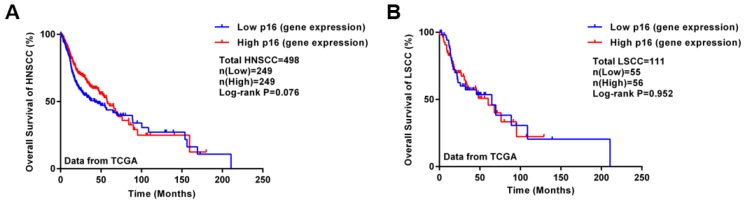
Survival analysis of HNSCC patients with different p16 levels in tumor samples. Kaplan-Meier analysis involved using transcriptome data for HNSCC and LSCC from the TCGA database (updated on Sept. 6, 2018). A: Kaplan-Meier plot of HNSCC patients with different p16 levels (498 samples); B: Kaplan-Meier plot of LSCC patients with different p16 levels (111 samples). High or low gene expression was classified by the median of p16 level.

For regulating the cell cycle, p16 protein is an inhibitory protein of the cell cycle-dependent kinase CDK4, which can prevent cell proliferation, also known as a negative regulation protein of the cell cycle. The latest National Comprehensive Cancer Network (NCCN) guidelines for 2018 (v2.2018 Head and Neck Cancers) distinguishes treatments for oropharyngeal cancer between p16 (HPV)-positive and p16-negative cancers. Therefore, the association between p16 protein and HPV-related laryngeal cancers is also worth investigating. The expression of p16 protein is slightly lower in laryngeal carcinoma than benign laryngeal lesions and significantly lower than in vocal polyps 44. Meanwhile, Hernandez et al.^45^ found that less than 10% of laryngeal tumors expressed p16, with only a small proportion (2%) of p16 and HPV DNA positivity in laryngeal cancer, so p16 expression was not closely related to HPV DNA status. As well, the correlation between p16 expression and HR-HPV DNA presence was not as strong as in cervical neoplastic lesions; some other factors may also cause increased p16 expression 46. Hence, p16 may not be a reliable alternative to HPV status in laryngeal cancer. Wittekindt et al.^47^ pointed out a poor correlation between p16 level and laryngeal cancer outcome. A study of laryngeal cancer patients in Hong Kong showed consistent results 48. Therefore, current studies suggest that p16 detection is not feasible as a surrogate marker for HPV infection in laryngeal cancer. In addition, Kaplan-Meier analysis using transcriptome data from The Cancer Genome Atlas database (TCGA) showed no significant difference in survival between high and low p16 expression groups with head and neck squamous cell carcinoma and laryngeal carcinoma (Figure 2). Although p16 plays crucial role in oropharyngeal cancer 49, the effects of p16 on other head and neck squamous cell carcinoma (HNSCC) such as LSCC remain unclear. However, most of the HNSCC samples in the TCGA database are LSCC and oral cavity carcinomas; the number of oropharyngeal cancer samples in the database is small.

## The detection of HPV

The pathogenicity of HPV is closely related to its type, so detection and classification of HPV has important clinical significance in screening, prevention and treatment of tumors and determining prognosis. Because HPV cannot be cultured *in vtro*, the diagnosis and typing of HPV cannot be performed by simple serological detection. Currently, the detection and typing of HPV mainly relies on molecular biological methods, including cytological methods, immunohistochemistry (IHC), *in situ* hybridization (ISH), western blot analysis of protein, and polymerase chain reaction (PCR). Table 2 summarizes conventional methods of HPV detection at DNA, mRNA, and protein levels.

### PCR and real-time PCR (RT-PCR)

PCR can detect expression in many samples with its advantages of being simple to perform, rapid, sensitive and low cost. In addition to using specific typing primers to detect a single type of HPV gene, general primers such as MY11 and MY09 can identify the conserved sequences of most HPV L1 genes. Nevertheless, cross contamination between samples easily occurs and high false-positive rates are possible. In addition, PCR is not a reliable method for HPV genotyping. RT-PCR can detect the mRNA level of HPV E6/E7 and accurately reflect the transcription activity of viral oncogenes. It can avoid the defect that the presence of HPV DNA detected by conventional PCR does not mean that viral genes are expressed or are in the state of transcription. As compared with other methods, RT-PCR, with its high sensitivity and specificity, can be used as the “gold standard” for HPV-related tumors to some extent 50. However, because it requires technical expertise and detection in fresh frozen tissue, its application is limited.

### *In situ* hybridization of nucleic acids

Nucleic acid hybridization, which has good specificity and sensitivity, can also be used for HPV typing, so it plays a certain role in studying the impact of different HPV types on the occurrence and development of diseases. As compared with DNA ISH based on DNA level, RNA ISH is more sensitive and specific. The sensitivity of DNA ISH is limited, especially for tumor samples with low viral copy numbers. Moreover, results of DNA ISH can be difficult to interpret in cases when hybridization signals are limited (faint, small, or non-uniformly distributed in tumor tissues) 51.

Development and application of the RNAscope technology solved some technical problems of RNA ISH 52, and the innovative RNA ISH method can be used to visualize individual RNA molecules in each cell. RNAscope can be used with a variety of sample types, including formalin-fixed paraffin-embedded tissue, fresh frozen tissue, fixed frozen tissue, and tissue microarray and cell samples. For example, RNAscope technology was used for detecting HPV in fresh frozen cervix tissues with low-grade squamous intraepithelial lesions 53 and evaluating a specific T-lymphocyte subset in paraffin specimens of non-small-cell lung cancer 54. Importantly, RNAscope technology has obvious advantages for detecting long non-coding RNAs and gene products that are little effective for antibodies 55-57.

As compared with conventional RNA ISH, RNAscope has obvious advantages for detecting HPV infection in laryngeal cancer because of its high sensitivity and specificity, the visualization and quantitative analysis of single molecules, and being able to detect partially degraded RNA 52. However, because of its high cost, it is mainly used in scientific research activities, with few clinical detection applications. The ideal detection method for HR-HPV with active transcription should be highly sensitive and specific, cost-effective and easy to use. There are great differences in HPV detection methods and no consensus has been reached 41.

### Detection of HPV at the protein level

For protein level detection, tumor suppressor protein p16 immunohistochemical staining is the main marker to evaluate the status of HPV infection. Positivity for p16 is a good prognostic marker of HPV-associated oropharyngeal squamous cell carcinoma 58, but contrary to the accepted pathogenic and prognostic role of HPV in oropharyngeal malignancies, its role in laryngeal cancer is relatively limited 59. Therefore, this method is not suitable for detecting the infection status of HPV in laryngeal cancer. Also, although IHC can detect virus antigens easily, it has some disadvantages such as false negatives and low sensitivity. Meanwhile, the positive detection rate of IHC may be much lower than that of PCR 60. Thus, IHC is not an ideal testing platform for HPV. In addition, western blot analysis detects proteins with antibodies. At present, this method is rarely used in HPV detection.

## Discussion

Emerging studies indicated that HPV infection may plays an important role in the initiation and progression of laryngeal cancer, but the clinical data for HPV infection in laryngeal cancer are largely inconsistent. In addition to genotypes of HPV infection being different, the infection rate varies among regions. Particularly, studies from China show high variation in HPV prevalence, from 2.4% to 76.42% (Table 1). Genetic diversity, environmental conditions, lifestyle, and the HPV detection method might explain the differential HPV infection rates in LSCC in different regions. The prevalence of HPV, particularly the HPV16 or HPV16/18 subtype, is high in Chinese LSCC patients as compared with those in Europe and North America 61, so people in different regions may have specific subtypes of HPV infection.

In this study, we reviewed recent advances about the correlation between HPV and laryngeal cancer, and summarized the potential carcinogenic mechanism of HPV infection from the levels of DNA, mRNA and protein: 1) the integration of the HPV DNA or genes into host cells; 2) carcinogenesis via activation of proto-oncogenes and inactivation of tumor suppressor genes; and 3) HPV viral proteins being indirectly carcinogenic via other biological progresses (cell apoptosis, cell cycle, etc.). We also summarized the common methods of HPV detection and analyzed their advantages and disadvantages.

As one of the most common malignant tumors in head and neck, laryngeal cancer seriously affects patients' quality of life and survival. HR-HPV infection is closely related to the occurrence and development of laryngeal cancer, but the carcinogenesis of HPV for laryngeal cancer has not been fully explained. Therefore, we must deeply study the molecular mechanism of laryngeal cancer caused by HPV infection. At the same time, rapidly detecting the type and degree of HPV infection by convenient and sensitive testing methods is crucial for preventing and treating laryngeal cancer.

Up to now, most studies at the DNA level have focused on the abnormal expression of various proto-oncogenes and tumor suppressor genes caused by HPV infection when explaining the role of oncogenic viruses in the development of laryngeal cancer. They rarely analyzed the possible interaction between two or more types of viruses. Some studies have shown that the incidence of laryngeal cancer is closely related to Epstein Barr virus (EBV) infection and pointed out that HR-HPV infection plays a certain role in inducing laryngeal cancer 19. In recent years, although a few studies 27 have examined the infection frequency of HPV and EBV in LSCC tissue samples, the role of the different HR-HPV genotypes and EBV in the etiology of LSCC has not been further studied. One study showed a significant correlation between tumor size and lymph node metastasis with combined versus single infection 62. Lymph node metastasis is the main metastasis of laryngeal cancer. Therefore, in laryngeal cancer, the role of HPV and EBV coinfection must be examined in the carcinogenic transformation of laryngeal epithelial cells.

The mRNA level of HPV is correlated with the occurrence and development of laryngeal cancer, but we have relatively few studies at this level. In comparison, in recent years, many reports have described the effects of non-coding RNA on the development and prognosis of laryngeal cancer. The microRNAs miR-497, miR-181a, and miR-155 and long noncoding RNA HOXAll-AS can affect the occurrence, development and prognosis of laryngeal cancer by changing the biological behaviors of laryngeal cancer cells, such as invasion, migration, proliferation and apoptosis 63-66. However, whether HR-HPV infection, which is closely related to laryngeal cancer, plays a role and whether it affects the expression of these non-coding RNA in laryngeal cancer is unknown.

In recent years, studies have shown that factors such as smoking may be a contributing factor to cancer-caused HPV infection. Patients who smoked frequently showed increased carcinogenicity of HPV in their bodies, which promoted the development of head and neck tumors 67. Meanwhile, the carcinogenic effect of tobacco may have a synergistic effect with HPV infection, and the viral cell replication may amplify the mutagenesis and metaplasia caused by smoking 68. Therefore, the study of the association between HPV infection and risk factors such as tobacco and alcohol abuse may be a future research direction. Another future research direction is whether HPV vaccine, which is widely promoted, has a certain impact on the occurrence and development of HPV-related laryngeal cancer.

## Figures and Tables

**Table 1:** Selected research on the prevalence of HPV in LSCC

Source	Publication date	Country, region	Sample size	Detection methods	HPV+ (%)	Genotypes
Ma, et al.^8^	1998	Northeast China (Shenyang)	102	PCR, Southern blot	58.8%	16, 18, 33, 6, 11
Peng, et al.^9^	2009	Southwest China (Chongqing)	123	PCR	76.42%	16, 18, 33, 45, 31, 52
Liu, et al.^10^	2010	Central China (Beijing)	84	qRT-PCR	34.5%	16, 18
Yao, et al.^11^	2010	Central China (Shanxi)	40	RT-PCR, IHC	57.5%	16, 18
Wang, et al.^12^	2011	Central China (Beijing)	84	PCR, ISH	36.9%	16, 18
Lu, et al.^13^	2012	East China (Shandong)	57	PCR	7.02%	16, 11, 43
Wu, et al.^14^	2012	East China (Shanghai)	46	PCR	6.52%	16, 11
Wang, et al.^15^	2014	South China (Hainan)	50	PCR	NA	16, 18
Wang, et al.^16^	2014	South China (Guangdong)	163	PCR	9.8%	NA
Guan, et al.^17^	2015	East China (Shanghai)	31	PCR	19.4%	16
Lu, et al.^18^	2016	South China (Guangdong)	82	PCR	2.4%	16
Zhang, et al.^19^	2017	Northwest China (Ningxia)	101	PCR	13.86%	16, 51, 58, 45, 67, 40, 84
Tong, et al.^20^	2018	Northeast China (Heilongjiang)	211	PCR, ISH	62.6%	16, 18, 11, 58, 13
Rodrigo, et al.^21^	2015	Northern Spain	62	PCR	1.6%	16
Atighechi, et al.^22^	2016	Iran	44	PCR	25%	16, 18, 6
Erkul, et al.^23^	2017	Istanbul, Turkey	78	Genotyping Assay RT-PCR	26.02%	16, 59
Shaikh, et al.^24^	2017	Bangladesh	NA	Nested PCR, automated DNA sequencing	21%	16, 33, 31
Kariche, et al.^25^	2018	Algeria	42	NA	2.38%	6
Milad, et al.^26^	2018	Egypt	56	PCR	3.6%	NA
Vazquez-Guillen, et al.^27^	2018	Mexico	195	PCR	47.7%	11, 52

ISH, *in situ* hybridization

**Table 2:** Common detection methods for HPV

	Detection method	Advantages	Disadvantages
DNA level	PCR	Simple, quick Sensitive Highly specific	Easy to cross-contamination between samples High false positive rate Perform HPV typing operations is tedious
	DNA ISH	High specificity Can be used for localization detection	Detection is affected by the DNA content in the specimen Results are sometimes difficult to explain
mRNA level	RT-PCR	High sensitivity High specificity Accurately reflects the transcription activity of viral oncogenes	Requires technical expertise Testing fresh frozen tissue
	RNA ISH	High sensitivity High specificity To some extent, it can be used as the "gold standard" for HPV-related tumors	The experimental operation requires high experience Poor repeatability
Protein level	p16 immuno- histochemistry	High sensitivity Strong prognostic indicators	Low specificity Mainly used for the detecting HPV in oropharyngeal cancer
	IHC	Virus antigen detection Easy to perform and locate	False-negative findings and low sensitivity
	Western blot analysis	NA	NA

IHC, immunohistochemistry; ISH, *in situ* hybridization

## References

[1] Almadori G, Bussu F, Cadoni G, Galli J, Paludetti G, Maurizi M (2005). Molecular markers in laryngeal squamous cell carcinoma: towards an integrated clinicobiological approach. Eur J Cancer.

[2] Tsimplaki E, Argyri E, Sakellaridis A, Kyrodimos E, Xesfyngi D, Panotopoulou E (2017). Oropharyngeal and laryngeal but not oral cancers are strongly associated with high-risk human papillomavirus in 172 Greek patients. J Med Virol.

[3] Li X, Gao L, Li H (2013). Human papillomavirus infection and laryngeal cancer risk: a systematic review and meta-analysis. J Infect Dis.

[4] Halec G, Holzinger D, Schmitt M (2013). Biological evidence for a causal role of HPV16 in a small fraction of laryngeal squamous cell carcinoma. Br J Cancer.

[5] Hobbs CG, Sterne JA, Bailey M, Heyderman RS, Birchall MA, Thomas SJ (2006). Human papillomavirus and head and neck cancer: a systematic review and meta-analysis. Clin Otolaryngol.

[6] Zhang C, Deng Z, Chen Y, Suzuki M, Xie M (2016). Is there a higher prevalence of human papillomavirus infection in Chinese laryngeal cancer patients? A systematic review and meta-analysis. Eur Arch Otorhinolaryngol.

[7] Xu Y, Liu S, Yi H (2014). Human papillomavirus infection in 674 Chinese patients with laryngeal squamous cell carcinoma. PLoS One.

[8] Ma XL, Ueno K, Pan ZM, Hi SZ, Ohyama M, Eizuru Y (1998). Human papillomavirus DNA sequences and p53 over-expression in laryngeal squamous cell carcinomas in Northeast China. J Med Virol.

[9] Peng FY, Jiang HR J, Liu F (2009). Detection and sequencing of different subtypes of HPV in laryngeal carcinoma. Journal of Chongqing Medical University.

[10] Liu B, Lu Z, Wang P, Basang Z, Rao X (2010). Prevalence of high-risk human papillomavirus types (HPV-16, HPV-18) and their physical status in primary laryngeal squamous cell carcinoma. Neoplasma.

[11] Yao J, Liu T (2010). Expression of human herpesvirus 6 and human papillomavirus 16/18 in the laryngeal squamous cell carcinoma and their relations. Journal of Shanxi Medical University.

[12] Wang P, Rao X, Li Y, Ning T, Liu G (2011). Detection of high-risk human papillomavirus types-16 and-18 in laryngeal squamous cell carcinoma. Cancer Research and Clinic.

[13] Lu G, Wang L, Sun Y, Li W, Hua H, Ge R (2012). Detection of different subtypes of HPV DNA in 57 cases of laryngeal carcinoma in easter of the shandong province. Journal of Otolaryngology and Ophthalmology of Shandong University.

[14] Wu J, Zhou Q (2012). Detection of different type of human papillomavirus in benign, premalignant and malignant lesions of the larynx. Journal of Modern Oncology.

[15] Wang H, Mu Z, Zhou X, Zhou X (2017). Relationship between high risk human papillomavirus E7 mRNA and laryngeal lesions. Chinese journal of gerontology.

[16] Wang H, Sun R, Hu W (2014). Human papillomavirus infection and HRAS, PIK3CA mutations analysis in patients with early laryngeal carcinoma. Cancer Research and Clinic.

[17] Guan L, Sun N, Sun G (2015). Analysis of subtypes of HPV infection in laryngeal squamous cell carcinoma and precancerous lesions and its clinical significance. Journal of Clinical Otorhinolaryngology Head and Neck.

[18] Lu Z, Li Y, Xu M (2016). The infection and integration status of high-risk human papillomavirus 16/18 in laryngeal cancer. The Journal of Practical Medicine.

[19] Zhang Y, Chen X, Li X (2017). Correlation of positive expressions of HPV and EBV with laryngeal carcinoma. The Journal of Practical Medicine.

[20] Tong F, Geng J, Yan B (2018). Prevalence and Prognostic Significance of HPV in Laryngeal Squamous Cell Carcinoma in Northeast China. Cell Physiol Biochem.

[21] Rodrigo JP, Hermsen MA, Fresno MF (2015). Prevalence of human papillomavirus in laryngeal and hypopharyngeal squamous cell carcinomas in northern Spain. Cancer Epidemiol.

[22] Atighechi S, Meybodian M, Dadgarnia MH, Baradaranfar MH, Behniafard N (2016). Investigating the Prevalence of Human Papilloma Virus in Squamous Cell Carcinoma of the Larynx and Its Correlation with Disease Prognosis. Iran J Otorhinolaryngol.

[23] Erkul E, Yilmaz I, Narli G, Babayigit MA, Gungor A, Demirel D (2017). The presence and prognostic significance of human papillomavirus in squamous cell carcinoma of the larynx. Eur Arch Otorhinolaryngol.

[24] Shaikh MH, Khan AI, Sadat A (2017). Prevalence and types of high-risk human papillomaviruses in head and neck cancers from Bangladesh. BMC Cancer.

[25] Kariche N, Hortal MT, Benyahia S (2018). Comparative assessment of HPV, alcohol and tobacco etiological fractions in Algerian patients with laryngeal squamous cell carcinoma. Infect Agent Cancer.

[26] Milad P, Kassamy H, Askoura A, Abuelela S, Salem R, Ragab D (2018). Prevalence of human papillomavirus in benign and malignant laryngeal lesions in Egyptian patients: Cross-sectional study. Clin Otolaryngol.

[27] Vazquez-Guillen JM, Palacios-Saucedo GC, Rivera-Morales LG (2018). Infection and coinfection by human papillomavirus, Epstein-Barr virus and Merkel cell polyomavirus in patients with squamous cell carcinoma of the larynx: a retrospective study. PeerJ.

[28] Syrjanen K, Syrjanen S, Pyrhonen S (1982). Human papilloma virus (HPV) antigens in lesions of laryngeal squamous cell carcinomas. ORL J Otorhinolaryngol Relat Spec.

[29] Torrente MC, Rodrigo JP, Haigentz M Jr (2011). Human papillomavirus infections in laryngeal cancer. Head Neck.

[30] Ye Q, Zhao S, He J, Qiang B, Ying X (2007). Protein expression of HPV16 and its early gene E6, E7 in human laryngeal squamous cell carcinoma and its significance. Journal of Second Military Medical University.

[31] zur HH (2002). Papillomaviruses and cancer: from basic studies to clinical application. Nat Rev Cancer.

[32] Feng Y, Zhang X, Zhang J, Li Y, Luo K (2004). Analysis of the correlation of early laryngeal carcinoma with HPV infection and expressions of p53, p16 and nm23 genes. Journal of China-Japan Friendship Hospital.

[33] Kiyono T, Foster SA, Koop JI, McDougall JK, Galloway DA, Klingelhutz AJ (1998). Both Rb/p16INK4a inactivation and telomerase activity are required to immortalize human epithelial cells. Nature.

[34] Veldman T, Horikawa I, Barrett JC, Schlegel R (2001). Transcriptional activation of the telomerase hTERT gene by human papillomavirus type 16 E6 oncoprotein. J Virol.

[35] Branca M, Giorgi C, Santini D (2005). Survivin as a marker of cervical intraepithelial neoplasia and high-risk human papillomavirus and a predictor of virus clearance and prognosis in cervical cancer. Am J Clin Pathol.

[36] Fusconi M, Campo F, Gallo A (2017). Laryngeal Cancer, HPV DNA vs E6/E7 mRNA Test: A Systematic Review. J Voice.

[37] Syrjanen S (2005). Human papillomavirus (HPV) in head and neck cancer. J Clin Virol.

[38] Wang H, Zhou X, Huang J, Zhou X (2017). Detection and clinical significance of mRNA expression of human papillomavirus E7 and tumor proliferation and migration related genes in elderly laryngeal cancer tissues. Chinese Journal of Gerontology.

[39] Poltronieri P, Sun B, Huang KY, Chang TH (2018). State-of-the-Art on Viral microRNAs in HPV Infection and Cancer Development. Microrna.

[40] Stasikowska-Kanicka O, Wagrowska-Danilewicz M, Danilewicz M (2011). Effect of human papillomavirus on cell cycle-related proteins p16INK4A, p21waf1/cip1, p53 and cyclin D1 in sinonasal inverted papilloma and laryngeal carcinoma. An *in situ* hybridization study. Folia Histochem Cytobiol.

[41] Zeng H, Yang H, Li W, Tu X, Kong W (2017). Clinical significance of abnormal expression of specific protein in laryngeal. Hainan Medical Journal.

[42] Krecicki T, Smigiel R, Fraczek M, Kowalczyk M, Sasiadek MM (2004). Studies of the cell cycle regulatory proteins P16, cyclin D1 and retinoblastoma protein in laryngeal carcinoma tissue. J Laryngol Otol.

[43] Scheel A, Bellile E, McHugh JB (2016). Classification of TP53 mutations and HPV predict survival in advanced larynx cancer. Laryngoscope.

[44] Li Q, Qin X, Ye X, Gu Li, Zhu X (2017). Expression of HPV-DNA and P16 protein in laryngeal cancer, benign laryngeal lesions, and vocal cord polyps. Chinese Journal of Clinical Medicine.

[45] Hernandez BY, Rahman M, Lynch CF (2016). p16(INK4A) expression in invasive laryngeal cancer. Papillomavirus Res.

[46] Laco J, Slaninka I, Jirásek M, Celakovský P, Vosmiková H, Ryska A (2008). High-risk human papillomavirus infection and p16INK4a protein expression in laryngeal lesions. Pathol Res Pract.

[47] Wittekindt C, Wuerdemann N, Gattenlöhner S (2017). The role of high-risk human papillomavirus infections in laryngeal squamous cell carcinoma. Eur Arch Otorhinolaryngol.

[48] Lam EWH, Chan MMH, Wai CKC (2018). The role of human papillomavirus in laryngeal cancer in Southern China. J Med Virol.

[49] Golusiński P, Pazdrowski J, Szewczyk M (2017). Is immunohistochemical evaluation of p16 in oropharyngeal cancer enough to predict the HPV positivity. Rep Pract Oncol Radiother.

[50] Leemans CR, Braakhuis BJ, Brakenhoff RH (2011). The molecular biology of head and neck cancer. Nat Rev Cancer.

[51] Bishop JA, Lewis JS Jr, Rocco JW, Faquin WC (2015). HPV-related squamous cell carcinoma of the head and neck: An update on testing in routine pathology practice. Semin Diagn Pathol.

[52] Wang F, Flanagan J, Su N (2012). RNAscope: a novel *in situ* RNA analysis platform for formalin-fixed, paraffin-embedded tissues. J Mol Diagn.

[53] Mills AM, Coppock JD, Willis BC, Stoler MH (2018). HPV E6/E7 mRNA *in situ* Hybridization in the Diagnosis of Cervical Low-grade Squamous Intraepithelial Lesions (LSIL). Am J Surg Pathol.

[54] Thommen DS, Koelzer VH, Herzig P (2018). A transcriptionally and functionally distinct PD-1+ CD8+ T cell pool with predictive potential in non-small-cell lung cancer treated with PD-1 blockade. Nat Med.

[55] Jin S, Yang X, Li J, Yang W, Ma H, Zhang Z (2019). p53-targeted lincRNA-p21 acts as a tumor suppressor by inhibiting JAK2/STAT3 signaling pathways in head and neck squamous cell carcinoma. Mol Cancer.

[56] Li J, Hao Y, Mao W (2019). LincK contributes to breast tumorigenesis by promoting proliferation and epithelial-to-mesenchymal transition. J Hematol Oncol.

[57] Nakajima T, Uehara T, Maruyama Y, Iwaya M, Kobayashi Y, Ota H (2016). Distribution of Lgr5-positive cancer cells in intramucosal gastric signet-ring cell carcinoma. Pathol Int.

[58] Barasch S, Mohindra P, Hennrick K, Hartig GK, Harari PM, Yang DT (2016). Assessing p16 Status of Oropharyngeal Squamous Cell Carcinoma by Combined Assessment of the Number of Cells Stained and the Confluence of p16 Staining: A Validation by Clinical Outcomes. Am J Surg Pathol.

[59] Combes JD, Franceschi S (2014). Role of human papillomavirus in non-oropharyngeal head and neck cancers. Oral Oncol.

[60] Azzimonti B, Hertel L, Aluffi P (1999). Demonstration of multiple HPV types in laryngeal premalignant lesions using polymerase chain reaction and immunohistochemistry. J Med Virol.

[61] Syrjänen S, Syrjänen K (2019). HPV in Head and Neck Carcinomas: Different HPV Profiles in Oropharyngeal Carcinomas-Why?. Acta Cytol.

[62] Drop B, Strycharz-Dudziak M, Kliszczewska E, Polz-Dacewicz M (2017). Coinfection with Epstein-Barr Virus (EBV), Human Papilloma Virus (HPV) and Polyoma BK Virus (BKPyV) in Laryngeal, Oropharyngeal and Oral Cavity Cancer. Int J Mol Sci.

[63] Niu JT, Liu SG, Huang YW, Li C (2018). The effect of miR-497 on laryngeal squamous cell carcinoma invasion through modulating PlexinA4. Zhonghua Er Bi Yan Hou Tou Jing Wai Ke Za Zhi.

[64] Zhao X, Zhang W, Ji W (2018). miR-181a targets GATA6 to inhibit the progression of human laryngeal squamous cell carcinoma. Future Oncol.

[65] Zhao X, Zhang W, Ji W (2018). YB-1 promotes laryngeal squamous cell carcinoma progression by inducing miR-155 expression via c-Myb. Future Oncol.

[66] Qu L, Jin M, Yang L (2018). Expression of long non-coding RNA HOXA11-AS is correlated with progression of laryngeal squamous cell carcinoma. Am J Transl Res.

[67] Wittekindt C, Wagner S, Mayer CS, Klussmann JP (2012). Basics of tumor development and importance of human papilloma virus (HPV) for head and neck cancer. Laryngorhinootologie.

[68] He J, Meng H, Yao G, Fu Y, Geng J (2017). New research progress of HPV and Larynx cancer. Progress in Modern Biomedicine.

